# Causes of neonatal and maternal deaths in Dhaka slums: Implications for service delivery

**DOI:** 10.1186/1471-2458-12-84

**Published:** 2012-01-26

**Authors:** Fatema Khatun, Sabrina Rasheed, Allisyn C Moran, Ashraful M Alam, Mohammad Sohel Shomik, Munira Sultana, Nuzhat Choudhury, Mohammad Iqbal, Abbas Bhuiya

**Affiliations:** 1 ICDDR,B, 68 Shaheed Tajuddin Ahmed Sarani, Mohakhali, Dhaka 1212, Bangladesh; 2 BRAC, BRAC Centre, 75 Mohakhali, Dhaka 1212, Bangladesh

## Abstract

**Background:**

Bangladesh has about 5.7 million people living in urban slums that are characterized by adverse living conditions, poor access to healthcare services and health outcomes. In an attempt to ensure safe maternal, neonatal and child health services in the slums BRAC started a programme, MANOSHI, in 2007. This paper reports the causes of maternal and neonatal deaths in slums and discusses the implications of those deaths for Maternal Neonatal and Child Health service delivery.

**Methods:**

Slums in three areas of Dhaka city were selected purposively. Data on causes of deaths were collected during 2008-2009 using verbal autopsy form. Two trained physicians independently assigned the cause of deaths.

**Results:**

A total of 260 newborn and 38 maternal deaths were identified between 2008 and 2009. The majority (75%) of neonatal deaths occurred during 0-7 days. The main causes of deaths were birth asphyxia (42%), sepsis (20%) and birth trauma (7%). Post partum hemorrhage (37%) and eclampsia (16%) were the major direct causes and hepatic failure due to viral hepatitis was the most prevalent indirect cause (11%) of maternal deaths.

**Conclusion:**

Delivery at a health facility with child assessment within a day of delivery and appropriate treatment could reduce neonatal deaths. Maternal mortality is unlikely to reduce without delivering at facilities with basic Emergency Obstetric Care (EOC) and arrangements for timely referral to EOC. There is a need for a comprehensive package of services that includes control of infectious diseases during pregnancy, EOC and adequate after delivery care.

## Background

High maternal and neonatal mortality rates are still major challenges facing Bangladesh. Despite significant reduction over the last two decades, the maternal mortality ratio and neonatal mortality rate are high at 194 per 100,000 live births and 37 per 1000 live births [1,2] respectively. The situation is worse in the country's urban slums with neonatal mortality rate of 43.7 per 1000 live births [3]. The main causes of maternal deaths are hemorrhage (31%), eclampsia (20%) according to 2010 National survey [1]. There is no information available related to maternal cause of death in the urban slums. There are no national surveys of exploring the causes of neonatal deaths in Bangladesh. However, a study conducted selected rural area reported birth asphyxia (45%), low birth weight/pre maturity (15%) and neonatal sepsis (12%) as the main causes of neonatal deaths [4].

Urban slums deserve special attention as they host over 5.7 million people, roughly 3.8% of the total national population [3]. Recent data show that most of the global urban growth is occurring in the low income countries of Asian cities such as Dhaka [5]. If one only considers the great population densities within the cities, cities clearly present a very different health environment than the more sparsely populated rural areas. Though rapid urbanization is driven mostly by a more dynamic economic environment in the cities, it also attracts many of the poorest and disadvantaged members of rural society [3]. This population presents an enormous challenge to the urban infrastructure, including public health and healthcare systems already stretched beyond capacity to meet the need of the present population. Key elements of urban infrastructure, including water and sanitation are already inadequate for the need of the population and effects of these inadequacies are likely to be felt at the level of population health [3]. The size of slum population and the unique challenges of providing health services to the residents of urban slums make urban health a major issue of concern for Bangladesh and other similar nations. Among the many challenges, designing appropriate service delivery programs, especially for reducing maternal and neonatal mortality, is critical on many counts. Reaching the slum dwellers with appropriate services in terms of service components and delivery strategy is essential. Service components should be effective in reducing mortality from common causes of deaths. Keeping this in mind, a study to collect causes of deaths of adult females and children < 5 years of age in slums was conducted between 2008 and 2009 as a part of a large scale maternal, neonatal and child health (MNCH) service delivery program MANOSHI (*Maa Nobojatok O Shishu *or Mother, Newborn and Child) implemented by BRAC [6]. This paper reports maternal and neonatal deaths in the study area and discusses their implications in the context of the existing and future possible service delivery programs in the urban setting.

## Methods

### Study area

This study was conducted in three selected areas of Dhaka city which contained slums: *Gulshan (Korail, Shat tola), Uttara(north and south Arichpur) *and *Kamrnagir Char *covering approximately 300,000 residents in total. All deaths among adult females and children < 5 years of age who were residents of the slums (based on program identification numbers) in the study area between January 2008 and December 2009 were eligible for the study.

To reduce morbidity and mortality of mothers, newborns and children in the urban slums of Bangladesh, BRAC started a slum-based MNCH program called MANOSHI [6]. Each BRAC delivery centre covered about 2000 households and was manned by two urban birth attendants (UBA) and one Community Health Worker (CHW). One community midwife oversaw the operations of 4-5 delivery centers. At the delivery centre, services such as normal delivery, post natal care (PNC), assessment of mothers and the neonate for identification of maternal and neonatal danger signs, referral when complications warranted and management and referral for birth asphyxia and low birth weight cases were provided. The program used CHWs to identify pregnancies, provide Ante Natal Care (ANC), provide counseling related to maternal and child health, bring mothers to slum-based delivery centers for normal delivery and provide appropriate referral linkages to secondary and tertiary facilities for delivery complications [6]. Further details about the staffing and design of MANOSHI can be found elsewhere [7].

### Identification of deaths

**Figure 1:**
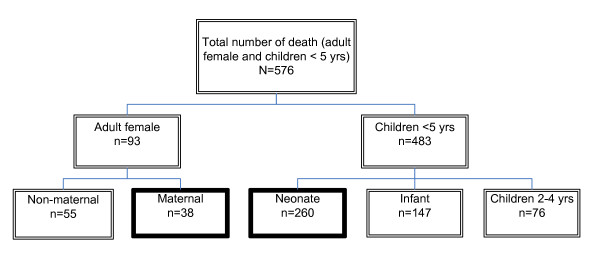
**Numbers of deaths by categories**.

In the three slums deaths of adult female and under five children were identified through the existing MANOSHI program Management Information System (MIS). Study team visited households to collect information on death. These visits were made monthly using a standard form. In addition, information on the deaths was collected from traditional birth attendants, drug sellers and local health providers. Once the final list was compiled, the households were visited to collect further information. The details of all the deaths investigated are provided in Figure 1.

In this paper we discussed the maternal and neonatal deaths. Maternal deaths was defined as any death of a woman (aged 15-49 years) while pregnant or within 42 days of delivery or termination of pregnancy, irrespective of the duration and site of the pregnancy, from any cause related to or aggravated by the pregnancy or its management, but not from accidental or incidental causes. Neonatal deaths were defined as death of newborn within 28 days of birth due to any cause.

### Data collection

After identification of death, a trained female interviewer visited the household to conduct a verbal autopsy interview within 15 to 30 days of the death. The verbal autopsy questionnaire used was based on those developed by INDEPTH (http://www.indepth-network.org) and WHO [8]. The questionnaire was adapted to local customs and culture and was translated into Bengali. The questionnaire included sections on background, events leading to the death, signs and symptoms of illness leading to the death, pregnancy history, care seeking behavior and perception of the respondents about the cause of death. We also collected descriptions of signs and symptoms of complications. A brief story of the events leading to the death was recorded. The story comprised of hospital records if available and of the description of the events given by the respondents. In addition, hospital records, laboratory tests and death certificates were photocopied and included in the review process. For verbal autopsy interviews of deceased women, we selected a close family member had been present during the illness that led to death, and was able to describe the symptoms and medical consultations prior to death. For newborn deaths, mothers or close relatives were the primary respondents. For neonatal deaths, 78% of the respondents were parents and for maternal deaths, 21% of respondents were husbands, 29% were parents and parents in laws, 26% were siblings, 5% were adult sons or daughters and 18% were neighbors of the deceased.

### Assigning causes of death

Two registered physicians independently reviewed each questionnaire and assigned a cause of death based on International Classification of Diseases version 10 (ICD 10) [9]. When there was a disagreement between the two reviewers, the verbal autopsy questionnaire was reviewed by a third physician. Final cause of death was assigned based on consensus of at least two physicians. If disagreement remained, the cause was assigned a "not determined" code. If there were not enough information to assign a definite cause, the code "unknown" was given by the physicians. After independent review by two physicians, 92% of assigned causes of death were concordant and for 8% of the cases arrived at jointly based on consensus of the two primary physicians.

### Quality assurance

#### Identification and verification of deaths

All deaths that were identified from MANOSHI program MIS and other local sources were verified by study team through household visits using a standard form. Duplication of deaths from different sources was avoided through using names of the household heads and MANOSHI program identification numbers.

#### Training of field workers

Three graduate female research assistants and 1 supervisor constituted the data collection team. The team was trained for two weeks on the study questionnaire and on how to deal with situation while conducting interviews on a sensitive issue such as deaths. The training included field testing. Physicians received two-week training on how to review the questionnaire in order to assign ICD 10 codes for cause of death.

#### Quality control of the data

For every 10 respondent questionnaires, a quality control field officer re-interviewed one respondent to verify data quality.

### Data analysis

For reporting purposes neonatal sepsis and pneumonia were combined to one category [10]. Descriptive analysis was done as appropriate. Statistical analyses were performed using Statistical Package for Social Sciences (SPSS, Chicago, IL) version 12.0.

### Ethical consideration

Ethical Review Committee of the ICDDR,B provided approval. Informed verbal consent was taken from all interviewees and confidentiality and anonymity were ensured.

## Results

**Figure 2:**
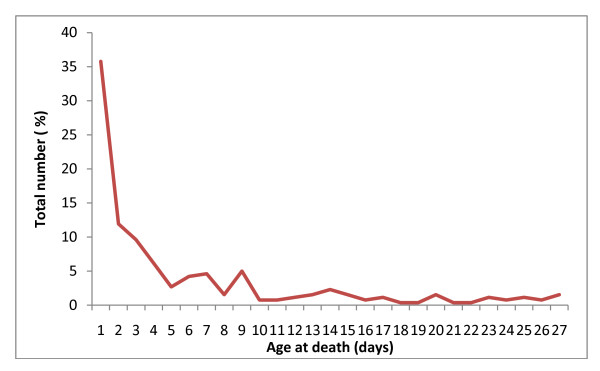
**Age of neonate at death**.

Between 2008 and 2009, there were 260 neonatal and 38 maternal deaths. Of the neonatal deaths, 36% occurred during the first day and 75% during the first week of life (Figure 2). Forty seven percent of neonate were low birth weight (< 2500 g) and 52% were pre-term (< 37 weeks). Thirty seven percent of mothers with a neonatal death had no education, 60% of mothers availed ANC from MANOSHI and 42% delivered at home (Table 1).

**Table 1:** Reproductive and socio-demographic characteristics of mothers of the deceased neonates (2008-9)

Socio-demographic characteristics	N = 260 (%)
***Mother's age***	

15-19y	52 (20.2)

20-24y	90 (35.0)

25-29y	68 (26.5)

30-35y	21 (8.2)

35-39y	19 (7.4)

> 40y	7 (2.7)

***Monthly household income***	

< 5000 Tk.(US$66)	124 (47.7)

5000-10000 Tk. (US$ 67-133)	120 (46.2)

> 10, 000Tk.(US$ 134)	16 (6.2)

***Mothers' education***	

None	95 (37.0)

1-5 years	107 (41.6)

6+years	55 (21.4)

***Mothers' occupation***	

Housewife	209 (80.4)

Maid	15 (5.8)

Garments worker	22 (8.5)

Others	14 (5.4)

**Reproductive characteristics of mothers**	

***Received ante- natal care***	

Yes	223 (85.8)

No	31 (11.9)

Unknown	6 (2.3)

***ANC taken from *(**n = 223)	

BRAC (CHWs)	157 (70.4)

MBBS doctors	36 (16.1)

Nurse, Midwives, CSBA	24 (10.8)

Drug sellers	2 (0.9)

Others	4 (1.8)

***Place of delivery***	

Home	108 (41.5)

BRAC delivery centre	63 (24.2)

Other facility	81 (31.2)

On the way to facility	5 (1.9)

Unknown	3 (1.2)

***Mode of delivery***	

Normal vaginal delivery	219 (84.6)

Caesarean Section (C/S)	35 (13.5)

Unknown	5 (1.9)

***Received post natal care***	

Yes	151 (58.8)

No	98 (38.1)

Unknown	8 (3.1)

***PNC taken from *(**n = 152)	

Skilled (MBBS, Nurse)	76 (50.0)

BRAC (CHWs)	70 (46.1)

Unskilled (village doctors, drug sellers CSBA. trained TBA)	6 (3.9)

***Birth order***	

1st	82 (31.5)

2nd	67 (25.8)

3rd	53 (20.4)

4th and above	58 (22.3)


**Figure 3:**
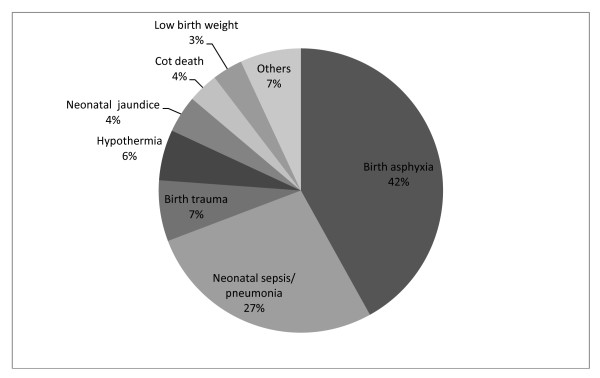
**Main causes of neonatal deaths (2008-9)**.

The three main causes of neonatal deaths were birth asphyxia (42%), neonatal sepsis and pneumonia (27%) and birth trauma (7%) (Figure 3). We were able to assign underlying cause for 60% of the deaths and found that the major underlying cause of neonatal deaths was low birth weight (74%) (Data not shown). We collected information about lay perception of cause of death. From lay beliefs, the leading reason for neonatal death was supernatural causes (evil spirit/evil eyes), believed by 22% of respondents, premature birth (9%) and other biomedical causes (Table 2).

**Table 2:** Lay perception regarding causes of deaths in neonatal period

Lay perception of cause of neonatal deaths	N (%)
Supernatural causes (Evil eye/spirit, *Alga batas*)	60 (23.1)

Premature birth	23 (8.8)

Cold	22 (8.5)

Low birth weight	16 (6.2)

Pneumonia	14 (5.4)

Wrong process of delivery	13 (5.0)

Other causes*	112 (43.1)

*Other causes included fever, jaundice, twin could not live alone, God knows, vomiting, unable to provide treatment, lack of good quality of treatment at hospital, experience of violence during pregnancy by mother

The median age of women who died due to maternal causes was 25 years. Of the women who died due to maternal causes, 42% did not receive any formal education, 82% received antenatal care, 86% were multi-gravida, 31% delivered at home, 10% delivered at BRAC delivery centre and 75% had normal vaginal delivery (Table 3).

**Table 3:** Reproductive and socio-demographic characteristics of deceased women in the study area during 2008-2009

Socio-demographic characteristics	N (%) (n = 38)
***Age at death***	

15-19y	3 (7.9)

20-24y	11 (28.9)

25-29y	9 (23.7)

30-35y	6 (15.8)

35-39y	7 (18.4)

> 40y	2 (5.3)

***Monthly household income***	

< 5000 Tk.(US$ 66)	23 (60.5)

≥ 5000 Tk. (US$ 67 and more)	15 (39.5)

***Education***	

None	16 (42.1)

1-5 years	10 (26.3)

6+years	9 (23.7)

Unknown	3 (7.9)

***Occupation***	

Housewife	30 (78.9)

Garments worker	4 (10.5)

Others	4 (10.5)

***Received ante-natal care***	

Yes	31 (81.6)

No	4 (10.5)

Unknown	3 (7.9)

***ANC taken from(n = 31)***	

BRAC (CHWs)	16 (51.6)

MBBS doctors	6 (19.4)

Unknown	9 (29.0)

***Place of delivery (n = 29)***	

Home	9 (31.0)

BRAC delivery centre	3 (10.3)

Government hospital	12 (41.4)

Other NGO/private hospital	5 (17.2)

***Mode of delivery(n = 28)***	

Normal vaginal delivery	21 (75)

Caesarean section (C/S)	5 (17.9)

Unknown	2 (7.1)

***Received post natal care (n = 28)***	

Yes	15 (49.5)

No	12 (31.6)

Unknown	11 (28.9)

**Figure 4:**
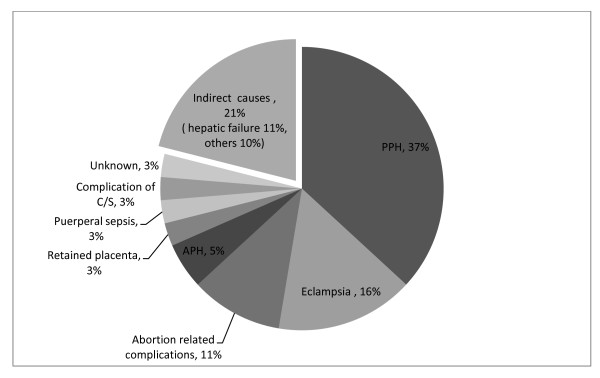
**Main causes of maternal deaths (2008-9)**.

The major causes of maternal deaths were post partum hemorrhage (PPH) (37%), eclampsia (16%), and hepatic failure (11%) (Figure 4). We were able to assign an underlying cause for 37% maternal deaths. The major underlying cause of maternal death was hepatic failure due to viral hepatitis (43%) (Data not shown). Respondents believed that maternal deaths were caused by various biomedical conditions (Table 4). From the lay perspective 19% of the maternal deaths occurred due to bleeding followed by 16% due to jaundice (Table 4). No supernatural causes were reported.

**Table 4:** Perception of key respondent regarding causes of maternal deaths

Lay perception of cause of maternal deaths	N (%)
Bleeding after delivery	7 (18.9)

Jaundice	6 (15.8)

Complication of delivery	4 (10.5)

Retained placenta	3 (7.9)

Convulsion	3 (7.9)

Other causes*	15 (39.4)

*Other causes included transfused blood was bad, difficult breathing, Caesarian section (C/S) done by unqualified doctor, abortion

## Discussion

National data on causes of maternal and newborn deaths are available [1-3,11]. However, mortality data for urban slums is not usually segregated. In describing the causes of neonatal and maternal deaths in selected slums of urban Dhaka, this paper will provide useful information for program planners who are involved in providing healthcare to ensure maternal, neonatal and child survival in urban slums. In addition, because the data were collected from a large-scale MNCH program area, an understanding may be gleaned of areas of focus where emphasis could reduce the number of neonatal and maternal deaths in similar programs.

### Neonatal deaths

In our study, birth asphyxia (42%) is the dominant cause of neonatal deaths. This finding was supported by verbal autopsy data from a rural area of Bangladesh and neighboring country Nepal [4,12]. The high proportion of birth asphyxia in the urban slums and rural Bangladesh compared to global average of 23% indicates a lack of appropriate resuscitation after birth and a lack of immediate referral to hospitals [13]. Factors that lead to delivery complications and results in birth asphyxia are well known [14], however, implementation of preventive measures to avoid asphyxia during perinatal period remains challenging in low income countries [15] and is likely exacerbated by living conditions in the slums. MANOSHI program trained and incentivized CHWs to identify birth asphyxia, to provide basic management through bag and mask, and to give referrals if asphyxiation of the newborn continues after basic resuscitation techniques are applied at the BRAC delivery centers [6]. However, 45% of all deliveries took place at home and BRAC made no provision for providing any trained personnel for home deliveries [16]. So, for the large number of home deliveries (45% of all deliveries) there are no trained personnel in the community to help with identification or timely referral for birth asphyxia. Thus the program is less likely to have significant impact on reduction of death from birth asphyxia for substantial number of cases remains out of program coverage. Training CHWs who would make home visits during delivery to identify and make referrals for birth asphyxia could help reduce deaths.

The second largest cause of neonatal deaths was neonatal sepsis including possible serious bacterial infection and pneumonia. This echoes the findings of a similar study in rural Bangladesh [4]. According to WHO IMCI guidelines neonatal infections should be treated with injectable antibiotics [10]. In several interventions in India and Bangladesh, CHWs were trained to recognize and manage suspected serious neonatal infections with injectable procaine penicillin and gentamicin [17,18] which resulted in 60% and 34% reduction in neonatal mortality respectively. MANOSHI CHWs were not trained to provide such interventions and therefore, it is unlikely such steep reduction in mortality can be expected from MANOSHI. The MANOSHI CHWs were trained to only identify cases of neonatal infections during PNC home visits and were given incentives for making referrals. The measures were undermined, however as, according to the MANOSHI midline survey [16], only 13% of mothers received PNC visits at 3-28 days which may have left many neonatal infections undetected. And, even for those mothers visited by MANOSHI workers for PNC, it is unclear whether accurate detection and referral for sepsis occurred. Therefore, to reduce deaths from neonatal infections future programs should find a way to first reach the neonates during the critical period of first day after delivery and then reliably provide treatment directly or access to treatment for neonatal infections.

Seventy-five percent of neonatal deaths in the study area occurred within 7 days after birth, a finding in concurrence with other studies from Bangladesh [4,19]. Other researchers have shown that the timing of visit by trained personnel is crucial for child survival and that receiving visit on the day of birth reduced the risk of neonatal mortality by two-thirds among neonates who survived the first day [20]. However, only 34% of mothers and neonates in the MANOSHI program area received PNC visit within a day of delivery [16]. Although the proportion of women receiving PNC increased with the MANOSHI intervention, better emphasis on PNC visits on the day of delivery with CHWs trained to provide management, treatment and referral has the potential to further reduce neonatal deaths.

In view of the limited access to trained healthcare providers that slum residents have [1] and the important role that community member's play in ensuring timely and appropriate medical referral [21], it is important to understand community perceptions of causes of neonatal deaths. Specifically researchers have related caregiver/guardian perceptions about the causes and severity of diseases to the seeking of timely and appropriate healthcare [22-24]. Our data show that one-fifth of respondent believe that the supernatural (the evil eyes/spiritual air) causes newborn deaths. This demonstrates that attempts by the MANOSHI program to educate mothers in slums about neonatal complications and appropriate referral have not adequately succeeded and that many more innovations and efforts are needed to change community perceptions and improve health seeking behavior.

### Maternal deaths

Maternal deaths remain a problem in Bangladesh despite recent surveys pointing to a 40% reduction in maternal deaths over the last decade [2]. PPH and eclampsia continued to be a leading causes of maternal deaths similarly indicated by two existing national surveys [2,25], which points to the need for continued attention in these problems.

The MANOSHI program has used a two pronged approach to reduce maternal deaths due to birth complications: a) mothers are encouraged to come to the BRAC delivery centers where trained Urban Birth Attendants (Trained TBAs) provide *misoprostol *to reduce hemorrhage, identify maternal complications during delivery and refer appropriately; and b) mothers and other household members have been provided with education about danger signs of pregnancy and delivery so that they can directly seek appropriate care with the help of CHWs if complications arise during home births. As 19% of the deliveries take place at the BRAC delivery center and 49% take place at home [16] with no CHW present, educating communities effectively about danger signs is crucial. It is also necessary to educate the communities and pregnant women to seek delivery services from skilled health personnel to prevent both maternal and early neonatal deaths. It is important to note that none of the family members described supernatural causes for maternal deaths [26], indicating that the community has received effective education. In addition, there was a 57% reduction in the proportion of home delivery from 2007 to 2009, and a subsequent increase in facility-based deliveries indicating that the MANOSHI program found ways to get people to seek care from trained providers [16]. Researchers have reported that in rural Bangladeshi community members believed that hemorrhage after delivery is normal [27], which could lead to non-recognition of excessive bleeding and delay in care seeking. In the future, MNCH programs could address this misconception by using a simple low cost delivery mat [28] that can help lay people to recognize excessive bleeding and could encourage them to seek immediate emergency obstetric care.

Hepatic failure due to viral hepatitis was found to be one of the major underlying causes for maternal death in our study. During the study period, a hepatitis E outbreak was observed in the slums of Dhaka [29]. Viral hepatitis increased risk of death by up to 20% during pregnancy [30]. Fecal contamination of drinking water is the most important cause of hepatitis E epidemic [31] and, therefore, improvements both in the supplying of safe water and in sanitation in the slums need to be addressed. The MANOSHI program focused on clean delivery and appropriate referral but limited its ability to be effective by not addressing detection of, and appropriate referral for, infection such as viral hepatitis during pregnancy. For future reductions of maternal mortality it may be important for MNCH programs to identify prevalent infections based on location and context and to at least have the CHWs identify and refer illness from these infections during pregnancy. MNCH programs could also provide information about disease epidemics to other public health departments for other (non-health specific) interventions to reduce disease prevalence among neonates and mothers.

### Strengths of the study

Our study provides data on the causes of neonatal and maternal death for urban Bangladeshi slum population; this data has not been previously available. With a vital event registration system lacking, we used verbal autopsies to identify the causes of death and ICD-10 codes to unify the identification of different health conditions. We conducted this research in MANOSHI program area and we were notified quickly about the deaths of mothers and neonates through existing program MIS.

### Limitations of the study

We relied on community reports of signs and symptoms of illness to classify medical causes of death. The study did not capture all deaths in the study area as some maternal and newborn deaths were missed due to migration after death and others may not have been identified due to interpersonal sensitivities. It is also likely early maternal deaths due to abortion or ectopic pregnancy were also missed. Further, the study was conducted in selected slums and the findings may not be representative of all slums.

## Conclusions and recommendations

We found birth asphyxia and sepsis to be the major causes of neonatal death. In the context of existing MNCH program such as MANOSHI, it is possible that ensuring clean delivery may be necessary but not sufficient to reduce neonatal mortality. With health systems a comprehensive package of services to ensure detection, management and referral for neonatal complication is needed within the mechanism providing safe delivery. Innovative approaches to improve access to essential health services for neonatal complications should be tried within the existing health systems to ensure sustainability. Further, much work is needed to educate community members about neonatal danger signs and to engage them in effective care-seeking behavior.

For maternal deaths we found that PPH is still the major cause of deaths. Our study shows that viral hepatitis is an important cause of deaths for urban slums. The MANOSHI program has emphasized clean delivery and appropriate referral, leading to educating the community and encouraging people to access healthcare facilities for maternal delivery complications. However, for creating a comprehensive healthcare for reducing maternal mortality, further innovation will be needed to increase access to healthcare during pregnancy and appropriate and timely referral.

Finally, the process of delivery should be made safe for the mother and neonate. To that end MNCH programs focused on safe delivery only will not achieve their full potential. A complete package of services from pregnancy through the neonatal period must be provided for the urban slums or for other such resource poor-settings. Considering the serious lack of infrastructure and projected growth of slum areas in Bangladesh and other countries, striving for a complete service package and innovative modes of delivery is crucial for achieving millennium development goals.

## Competing interests

The authors declare that they have no competing interests.

## Authors' contributions

FK and SR analyzed the data and drafted the paper; FK, MI and MSS assigned cause of death and provided reviews at different stages; MAA and ACM designed and implemented the study and provided intellectual input into the analysis; NC and MS provided technical expertise, input in interpreting the results; and AB provided overall supervision for design, analysis and drafting of the manuscript.

## Author's information

Fatema Khatun Assistant Scientist, Centre for Equity and Health Systems, ICDDR,B, 68, Shaheed Tajuddin Ahmed Sarani, Mohakhali, Dhaka-1212, Bangladesh; Email: http://kfatema@icddrb.org, Fax: +880-2-8826050

## Pre-publication history

The pre-publication history for this paper can be accessed here:

http://www.biomedcentral.com/1471-2458/12/84/prepub
